# Mediterranean Diet and White Matter Hyperintensity Change over Time in Cognitively Intact Adults

**DOI:** 10.3390/nu14173664

**Published:** 2022-09-05

**Authors:** Suhang Song, Alexandra M. Gaynor, Emily Cruz, Seonjoo Lee, Yunglin Gazes, Christian Habeck, Yaakov Stern, Yian Gu

**Affiliations:** 1 Taub Institute for Research in Alzheimer’s Disease and the Aging Brain, Columbia University, New York, NY 10032, USA; 2 Department of Health Policy and Management, College of Public Health, University of Georgia, Athens, GA 30602, USA; 3 Department of Psychiatry and Biostatistics, Columbia University, New York, NY 10032, USA; 4 Mental Health Data Science, New York State Psychiatric Institute, New York, NY 10032, USA; 5 Cognitive Neuroscience Division, Department of Neurology, Columbia University, New York, NY 10032, USA; 6 Gertrude H. Sergievsky Center, Columbia University, New York, NY 10032, USA; 7 Department of Psychiatry, Columbia University, New York, NY 10032, USA; 8 Department of Epidemiology, Joseph P. Mailman School of Public Health, Columbia University, New York, NY 10032, USA

**Keywords:** Mediterranean diet, white matter hyperintensity change, moderation

## Abstract

Current evidence on the impact of Mediterranean diet (MeDi) on white matter hyperintensity (WMH) trajectory is scarce. This study aims to examine whether greater adherence to MeDi is associated with less accumulation of WMH. This population-based longitudinal study included 183 cognitively intact adults aged 20–80 years. The MeDi score was obtained from a self-reported food frequency questionnaire; WMH was assessed by 3T MRI. Multivariable linear regression was used to estimate the effect of MeDi on WMH change. Covariates included socio-demographic factors and brain markers. Moderation effects by age, gender, and race/ethnicity were examined, followed by stratification analyses. Among all participants, WMH increased from baseline to follow-up (mean difference [follow-up-baseline] [standard deviation] = 0.31 [0.48], *p* < 0.001). MeDi adherence was negatively associated with the increase in WMH (β = −0.014, 95% CI = −0.026–−0.001, *p* = 0.034), adjusting for all covariates. The association between MeDi and WMH change was moderated by age (young group = reference, *p*-interaction[middle-aged × MeDi] = 0.075, *p*-interaction[older × MeDi] = 0.037). The association between MeDi and WMH change was observed among the young group (β = −0.035, 95% CI = −0.058–−0.013, *p* = 0.003), but not among other age groups. Moderation effects by gender and race/ethnicity did not reach significance. Greater adherence to MeDi was associated with a lesser increase in WMH over time. Following a healthy diet, especially at younger age, may help to maintain a healthy brain.

## 1. Introduction

A Mediterranean diet (MeDi) is a healthy dietary pattern with high consumption of whole cereals, fruit, legumes, vegetables, and nuts, moderate intake of dairy products and alcohol (red wine), and low consumption of foods rich in refined grains, saturated fat, and red meat [1,2,3,4]. Consumption of olive oil is high, while saturated fatty acid intake is low [4]. Greater adherence to MeDi has been shown to be associated with better cognition [5,6] and reduced risks of mild cognitive impairment [7] and Alzheimer’s disease and its related dementias (ADRD) [1,8,9,10,11]. One possible mechanism could be that MeDi may exert a protective effect on brain structural status [2,12] and brain vascular function [1,3,13,14,15]. Emerging evidence supports the beneficial role of MeDi and other dietary factors in maintaining structural brain health, such as brain volume, cortical thickness, and white matter tract integrity [2,12]. However, less is known about the association between diet and cerebrovascular diseases, which are increasingly recognized as one of the key pathological features of ADRD [1,3,13,14,15].

White matter hyperintensity (WMH) burden seen in brain magnetic resonance imaging (MRI) is a cerebral small vessel disease [16,17,18] and has been shown to predict faster cognitive decline in older adults [17,18,19,20,21,22,23,24,25,26,27,28]. Thus, understanding the potential association between diet and WMH burden may clarify the pathway and further facilitate the prevention or delay of the onset of cognitive decline. However, previous evidence of the association between MeDi and WMH is scarce and inconsistent [3,9,14,15,29,30]. Two prior studies found that greater adherence to MeDi was significantly associated with lower WMH volume [3,30], while other studies reported that such an association did not reach statistical significance [9,14], even though all the studies focused on older adults and the WMH volume were measured similarly. Besides, few studies examined the role of MeDi on the longitudinal change in WMH burden; yet, once WMH starts to accumulate, it may be difficult to reverse the trend [31,32,33,34,35,36,37]. Thus, identifying a modifiable lifestyle factor, such as MeDi, which may slow the increase in WMH, may shed light on maintaining brain status. Moreover, the majority of the previous studies included older adults only [3,9], even though increases in WMH may begin to be observed in middle age [36]. Since it remains unclear when in the lifespan is the best time to begin following a healthy diet to prevent future brain aging, examining the MeDi-WMH change association in both young and older adults may assist in clarifying the start time to adhere to a healthy dietary pattern.

The current study aimed to examine whether greater adherence to MeDi is associated with less accumulation of WMH over time in a cohort of young, middle-aged, and older adults in the New York City metropolitan area.

## 2. Materials and Methods

### 2.1. Participants

Participants aged 20–80 years were derived from 2 ongoing studies (the Cognitive Reserve (CR) study and the Reference Ability Neural Network (RANN) study), which were conducted at Columbia University Irving Medical Center [38,39]. Participants of these 2 studies were recruited with the same eligibility criteria, underwent similar research procedures, and had similar demographic characteristics. The initial eligibility of the participant selection included right-handed, English speaking, no psychiatric or neurological disorders, and normal or corrected-to-normal vision. Further, the eligible participants were scanned by MRI and completed socio-demographic information and food frequency questionnaire (FFQ). In order to include only cognitively intact adults, those with a Dementia Rating Scale (DRS) score <130 were excluded. More detailed information was provided in previous reports in our lab [39,40].

**Figure 1:**
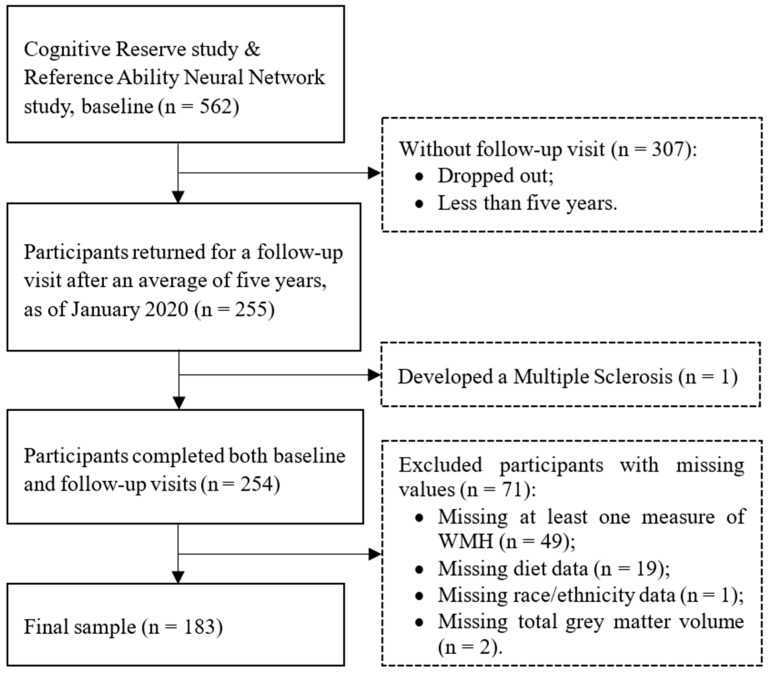
Flow chart of participant selection.

At baseline, 562 participants were enrolled in the 2 studies. As of January 2020, 255 participants returned for a follow-up visit after an average of 5 years from their baseline visit. One participant developed Multiple Sclerosis at the follow-up visit and was excluded. A total of 307 participants either dropped out or have not yet been seen for the follow-up visit. Among the 254 participants who completed both baseline and follow-up visits, we further excluded 49 participants lacking at least one measure of WMH, 19 missing diet data, 1 missing value of race/ethnicity, and 2 missing values of total grey matter volume. Hence, the current analyses included 183 cognitively intact adults (Figure 1). The baseline socio-demographic characteristics of the 183 participants were not different from those who were excluded (Table S1). The studies were approved by the Institutional Review Board of the College of Physicians and Surgeons of Columbia University (approval codes: AAAQ8249 and AAAI2752). All participants provided written informed consent.

### 2.2. Measures

MeDi score was calculated based on diet information obtained from Willett’s self-reported semi-quantitative FFQ (Channing Laboratory, Cambridge, MA, USA), which includes 61 items in 11 food categories of Mediterranean dietary pattern and has been validated and widely used in the literature [41,42]. In a subset of the current study population, we performed a reproducibility analysis in 24 participants who completed 2 FFQ reassessments on average 4.95 (standard deviation [SD] = 2.5) months apart. Test-retest reliability for each of the 61 food items was calculated using single measure absolute agreement intraclass correlation coefficients (ICCs) based on 2-way mixed-effect analysis of variance with 95% confidence intervals (CIs). The MeDi score showed good reliability and so did most of the food group components of MeDi score, suggesting the dietary assessment can reasonably represent the participants’ habitual dietary behaviors. For each food category, the frequency of consumption in servings per month was approximately estimated as the number of servings that this food category was consumed per month and then assigned scores 0, 1, 2, 3, 4, and 5. For the consumption of food categories with more characteristic of a Mediterranean dietary pattern (i.e., non-refined cereals, potatoes, fruits, vegetables, legumes and nuts, fish, and olive oil), a higher score corresponded to a more frequent intake. For the consumption of food categories with less characteristic of a Mediterranean dietary pattern (i.e., poultry, red meat, and full-fat dairy products), the scores were assigned on a reverse scale, indicating that a higher score corresponded to a less frequent intake. For alcohol, a score of 5 was assigned for consumption of 1–2 servings per month; a score of 0 was assigned for consumption of more than 60 servings per month or for no consumption; scores 4 to 1 were assigned for consumption of 3–4, 5–14, 15–30, and 31–60 servings per month, respectively. Detailed information on scores for each food category is provided in Table S2. The total MeDi score was calculated as the sum of the scores in 11 food categories, ranging from 0–55 continuously, with a higher score indicating greater adherence [41]. MeDi scores were further classified into three groups, low, middle, and high adherence, based on tertiles of total MeDi scores.

All brain images were acquired on Philips Achieva 3T MRI. A fluid-attenuated inversion recovery (FLAIR) scan was acquired with the following parameters: 11,000 msec repetition time, 2800 msec echo time, 256 × 189 voxels in-plane resolution, 23.0 × 17.96 cm field of view, and 30 slices with slice-thickness/gap of 4/0.5 mm and processed through a fully automatic supervised machine learning technique [43]. This method uses a randomized decision trees algorithm called random forest for training the classifier, which has been shown to be superior to the support vector machine algorithm often used for segmenting WMH. The final segmentation is a probability map in (0, 1), which denotes the likelihood that a given voxel is hyperintense, allowing for the calculation per subject of a normalized effective WMH volume. Periventricular and deep hyperintensity accumulations are separated using a ventricular template. Processed WMH images were visually checked and corrected if voxels were erroneously identified as WMH. A T1-weighted structural brain image was additionally acquired for each subject using MPRAGE sequence (TE/TR: 3/6.5 ms; Field of view: 256 mm; Flip angle: 8°; In-plane resolution: 256 × 256 voxels; Slice thickness/gap: 1/0 mm; Slices: 180). Both baseline and follow-up WMH volume, baseline total grey matter volume (TGMV), baseline intracranial volume (ICV), and baseline mean thickness were extracted from structural T1 scans after parcellation using FreeSurfer v5.1 (http://surfer.nmr.mgh.harvard.edu/) (accessed on 30 August 2022) [36]. In order to adjust for head size, TGMV was regressed with ICV, and the residuals of TGMV were used in the analysis.

The outcome variable was five-year change in WMH burden. Both baseline and follow-up WMH volumes were log10(WMH+1)-transformed, and WMH change scores were calculated as follow-up log scores minus baseline log scores. According to a previous study in our lab, the age of 43 years was an inflection point at which the total volume of WMH started to increase with age [36], and the age of ≥65 years was commonly used to refer to the older group [44,45,46,47,48,49]. Thus, this study included a stratification analysis by age group (young: <43, middle-aged: 43–64, and older: ≥65 years).

In addition to baseline TGMV residuals and mean thickness, covariates also included age, gender, years of education, National Adult Reading Test-assessed Intelligence Quotient (NARTIQ), race/ethnicity, total daily energy intake, and WMH, which were all measured at baseline; as well as follow-up interval, which was calculated as the time interval between the baseline and follow-up visits in years. Gender was dichotomized with males as the reference group. Race/ethnicity was categorized into three groups: non-Hispanic white and other (as the reference group), non-Hispanic black, and Hispanic. All other covariates were continuous variables.

### 2.3. Statistical Analysis

Mean and SD were reported for continuous variables, and frequency and percent were reported for gender and race/ethnicity. Analysis of variance (ANOVA) for continuous variables and Pearson’s chi-square tests for categorical variables were conducted to compare participants’ characteristics among tertile MeDi groups. A one-sample *t*-test was used to examine if the change in WMH burden from baseline to follow-up equals zero over an average of five years. Multivariable linear regression was used to estimate the effect of MeDi on WMH change. Moderation effects by age (young: <43, middle-aged: 43–65, and older: ≥65 years), gender, and race/ethnicity (non-Hispanic White and other, non-Hispanic Black, and Hispanic) were examined by including the interaction term of moderator × MeDi into the models. Stratification analyses examining the effect of MeDi on WMH change were subsequently performed by each age, gender, and race/ethnicity group. Tertile MeDi group was also examined in the moderation and stratification analyses. Two-sided *p* < 0.05 indicated significance; interaction terms were considered statistically significant at *p* < 0.10.

## 3. Results

### 3.1. Characteristics of Study Population

Table 1 presents a descriptive summary of the participants’ characteristics. Among all participants, MeDi score ranged from 12 to 43 with an average of 28.20 (SD = 5.54). WMH burden significantly increased over the mean baseline to follow-up interval of 4.86 years (SD = 0.61) (mean difference [MD] [follow-up-baseline] [SD] = 0.31 [0.48], *p* < 0.001), and this significant accumulation in WMH was also observed within each MeDi group, age group, gender group, and race/ethnicity group. Participants in middle and high MeDi groups, compared to those in low MeDi group, were more likely to be females (*p* = 0.001) and to report a higher level of total daily energy intake (*p* = 0.016). The tertile MeDi groups did not differ in other socio-demographic characteristics, WMH, brain markers, or follow-up interval.

### 3.2. Association between MeDi and WMH Change

**Figure 2:**
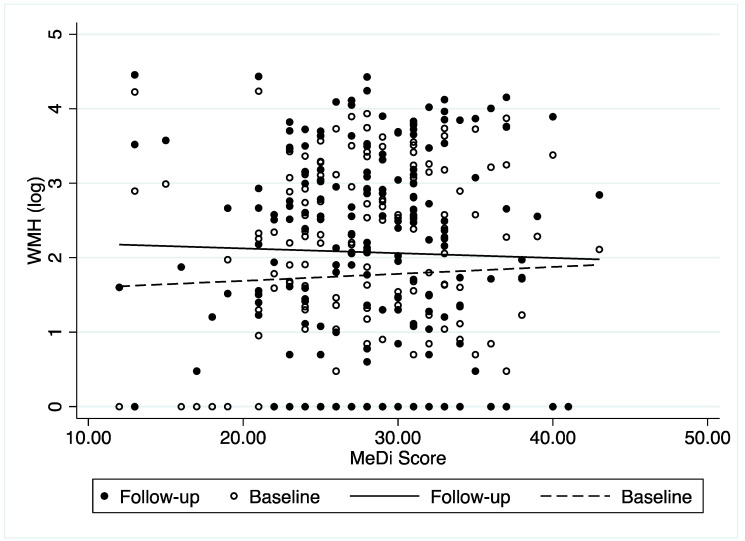
Relationship between Mediterranean diet score and white matter hyperintensity at baseline and follow-up visits.

Figure 2 shows the relationship between MeDi score and WMH (log) burden in baseline and follow-up visits. Among the majority of the participants, WMH burden in the follow-up visit was greater than or equal to that in the baseline visit. The change in the WMH burden between baseline and follow-up decreased with the increase in MeDi score. After adjusting for the effects of social-demographic characteristics, caloric intake, brain markers, and follow-up interval, we found that a higher MeDi score was associated with less of an increase in WMH burden (β = −0.014, 95% CI = −0.026–−0.001, *p* = 0.034), indicating that people with a healthier diet had less of an increase in WMH burden (Table 2), which was aligned with the findings presented in Figure 2.

### 3.3. Moderation Analysis on the Association between MeDi and WMH Change

After adjusting for the covariates, the association between MeDi and WMH change was significantly moderated by age (young group as the reference, *p*-interaction[middle-aged × MeDi] = 0.075, *p*-interaction[older × MeDi] = 0.037), such that young participants showed less increase in WMH burden for each unit increase in MeDi score, compared to the middle-aged or older participants (Table 2). Moderation effects by gender or race/ethnicity did not reach statistical significance.

**Figure 3:**
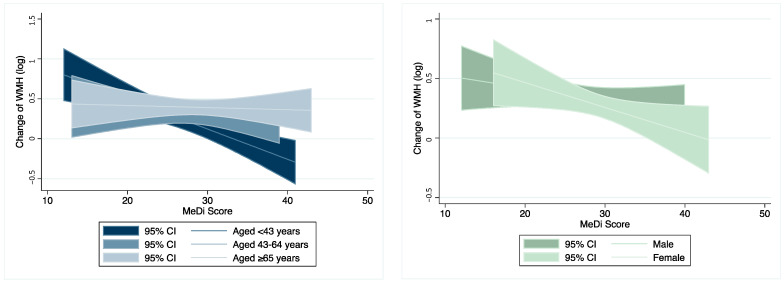

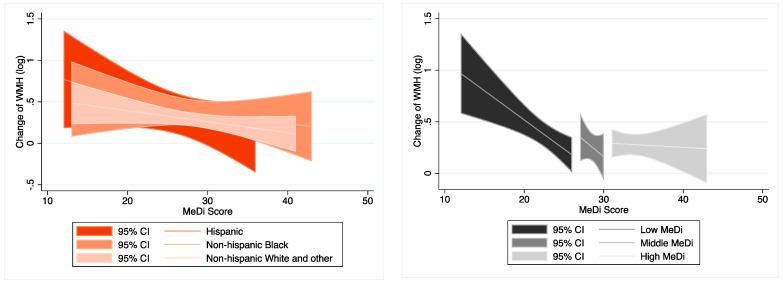
Relationship between Mediterranean diet score and change in white matter hyperintensity by age, gender, race/ethnicity, and tertile MeDi group.

In the stratification analysis by age, gender, and race/ethnicity, a significant association between MeDi score and WMH change from the fully adjusted model (Model 3) was observed in the young group (β = −0.035, 95% CI = −0.058–−0.013, *p* = 0.003), but such an association was not significant in other age, gender, or race/ethnicity groups (Table 2, Figure 3).

In terms of the three MeDi groups, in the fully adjusted model, the significant association between MeDi score and WMH change was observed in the low MeDi group only (β = −0.053, 95% CI = −0.091–−0.015, *p* = 0.008), indicating that participants with diets less similar to MeDi had less of an increase in WMH burden with each unit increase in MeDi score.

### 3.4. Association between Individual Food Categories and WMH Change

We further explored the associations between individual food categories and WMH change by entering the scores of the 11 food categories into the model simultaneously, adjusting for age and follow-up interval (Table 3). Greater intake of vegetables (β = −0.095, 95% CI = −0.162–−0.028, *p* = 0.006) and less intake of dairy (β = −0.045, 95% CI = −0.086–−0.004, *p* = 0.031) were significantly associated with less increase in WMH.

In order to test the moderation effect of age on the associations between individual food categories and WMH change, an interaction term of each individual food category by continuous age was added to the models, adjusting for follow-up interval. The interaction term of age and food category was significant in the models for vegetables (β = 0.003, 95% CI = −0.0002–0.007, *p*-interaction = 0.068), dairy (β = 0.002, 95% CI = −0.0001–0.004, *p*-interaction = 0.065), and alcohol (β = 0.003, 95% CI = 0.00005–0.005, *p*-interaction = 0.046), indicating that increasing age attenuated the associations between vegetable/dairy/alcohol intake and WMH change. In other words, associations between intake of these food types and WMH change were stronger among younger participants than older participants.

## 4. Discussion

In this longitudinal study of 183 cognitively intact adults, WMH burden increased within an average of five years from baseline to follow-up. Greater adherence to MeDi was associated with a lesser increase in WMH burden, and this association was moderated by age group and MeDi group. Specifically, a significant association was observed in the young group and the low MeDi group but not in other age and MeDi groups.

The finding that WMH burden increased over time has been commonly found in previous studies, the majority of which focused on older adults [37,50,51,52]. Indeed, WMH burden has been considered to reflect some age-related cerebrovascular diseases [53]. Only a few studies have measured WMH burden in middle-aged adults and reported that WMH burden commonly appeared in middle age [31,34,35,36]. Specifically, a previous cross-sectional study published in our lab reported an inflection point of 43 years, indicating that WMH burden started to accumulate after age 43 years [36]. Besides, another study found that WMH burden may also show an age-related increase among participants under 40 years, although the magnitude may be much smaller than in older people [31,32,33]. Overall, it seems WMH burden accumulates in all young, middle-aged and older adults.

Although a few studies examined the association between MeDi and cross-sectional WMH burden with both significant and insignificant findings [3,9,14,15,29,30] with only two studies reporting that MeDi was significantly associated with lower WMH burden [3,30], the association between MeDi and longitudinal change in WMH burden needs to be further investigated but has received extremely limited attention [54]. To the best of our knowledge, no study has focused on such an association, although one study reported a lesser increase in WMH burden with better omega-3 polyunsaturated fatty acids [54]. However, since it may be difficult to reverse the accumulation of WMH, once it starts to accumulate [31,32,33,34,35,36,37], the current study provides critical evidence that a modifiable lifestyle factor, MeDi, may slow the accumulation of WMH and help maintain brain status [3,30]. The biological mechanisms by which MeDi impacts WMH burden are unclear but might involve multiple pathways: [55] MeDi has been reported to be beneficial to improved endothelial function [56,57], lower obesity [58,59], and better cardiovascular health [14,55,60,61,62], by reducing the risks of cardiovascular disease [63,64], insulin resistance [65,66], and inflammation [67,68,69,70].

The significant impact of vegetables [12,71,72,73,74,75] and dairy [72,76,77,78] on WMH burden aligns with previous findings [30]. Some nutrients mainly found in vegetables, such as dietary fiber and vitamins B, C, D, and E, have been reported to be associated with white matter integrity [12,71,72,73,74,75]. Dairy products, which are rich in saturated fatty acids (SFAs), were reported to be associated with higher volume of white matter damage [72,76,77,78]. Fiber may help regulate the gut microbiota and glucose metabolism and further enhance insulin resistance in the brain [71]; B-vitamins may assist in regulating energy by supplying homocysteine metabolism [74]; vitamins C and E may provide antioxidant properties [73]; and vitamin D may be involved in the regulation of neurotransmitters and neurotrophin and may have anti-inflammatory and antioxidant neuroprotective capacities [75]. SFAs may be associated with metabolic, inflammatory, and microvascular changes, which may result in the damage of white matter in the brain [78].

Since few studies have examined how age moderates the association between MeDi and the increase in WMH, the current study provides innovative evidence on the moderation effect of age on this association. Notably, the results may demonstrate a relatively stronger association between diet and WMH change in the young group, despite baseline WMH burden being lower in this group, which might provide some evidence that it may be beneficial to following a healthy diet at an early age to maintain a healthy brain.

We found a significant moderation effect of the MeDi groups on the association between MeDi score and WMH change, wherein the association is stronger in the low MeDi group, which may result from diminishing marginal effects [79,80]. The contribution of MeDi to WMH change might be specified as increasing with diminishing marginal health effects until the adequacy cutoff level (i.e., somewhere in the middle MeDi group) is reached [79,80] and then the contribution of MeDi remains stable in high MeDi group.

This study is subject to several limitations. The first limitation is that the CR/RANN study only focused on the New York city metropolitan area, so the results may need to be generalized with caution. Second, the MeDi score was measured only one time per visit, even though the questions in the FFQ asked about the average frequency of food intake per month. People’s dietary patterns may change over time, so it may be cautious to interpret the answers in the FFQ as habitual dietary patterns. Third, other neurologic manifestations and brain alterations, such as celiac disease, may also need to be considered in further analyses [81]. Fourth, since the study is currently in the process of completing the five-year follow-up visit, the sample size of certain subgroups was limited, such as non-Hispanic black and Hispanic participants, which might account for the insignificant interaction between MeDi and race/ethnicity on WMH change. However, the study sample is not different from the overall study population, and the findings remained statistically significant after controlling for sufficient covariates. Finally, a wide age range was analyzed in the relatively small sample size, which may ignore smaller effects in the analyses.

This study also has several notable strengths. To begin with, this innovative study examined the association between MeDi and WMH change with a longitudinal study design, which may provide more convincing evidence of a true association, with less possibility of reverse causality, compared to cross-sectional studies. Furthermore, this study included both young and older adults, and therefore allowed for comparisons of the effect size of MeDi between young, middle-aged, and older adults. This study is also strengthened by using a valid and reliable FFQ measure with 11 food categories to calculate MeDi scores, which reflects a more comprehensive measure of overall dietary pattern. Besides, the scores for both overall MeDi (0–55) and each food category (0–5) were operationalized as large-scale scores, which readily allow for an estimation of dose-dependent associations with outcomes from a wider variation [3,41]. Lastly, we statistically controlled for the effects of structural brain measures and participants’ socio-demographic characteristics and demonstrated that the role of MeDi in WMH change could be detected independent of other potential confounders.

## 5. Conclusions

Greater adherence to MeDi was associated with reduced increase in WMH over time. Following a healthy diet, especially at an early age, may help maintain a healthy brain. The underlying mechanisms between MeDi and WMH accumulation warrant further examination.

## Figures and Tables

**Table 1:** Summary of characteristics (n = 183).

		All (n = 183)	Low MeDi (n = 67)	Middle MeDi (n = 50)	High MeDi (n = 66)	*p*-Value
MeDi score	Mean (SD)	28.20 (5.54)	22.54 (3.46)	28.54 (1.09)	33.68 (3.03)	<0.001 ***
	Range	12–43	12–26	27–30	31–43	
Follow up interval, years	Mean (SD)	4.86 (0.61)	4.97 (0.65)	4.90 (0.58)	4.73 (0.57)	0.060
Age, years	Mean (SD)	53.19 (16.52)	51.25 (16.75)	55.52 (15.71)	53.38 (16.9)	0.385
Age groups						
<43 years	n (%)	51 (27.87)	22 (32.84)	12 (24.00)	17 (25.76)	0.707
43–64 years	n (%)	72 (39.34)	27 (40.30)	20 (40.00)	25 (37.88)	
≥65 years	n (%)	60 (32.79)	18 (26.87)	18 (36.00)	24 (36.36)	
Education, years	Mean (SD)	16.33 (2.37)	16.34 (2.17)	16.38 (2.41)	16.27 (2.55)	0.969
NARTIQ	Mean (SD)	117.82 (8.20)	118.39 (7.80)	117.96 (8.91)	117.14 (8.12)	0.676
Calorie, kcal	Mean (SD)	1352.07 (557.11)	1237.48 (484.98)	1304.29 (518.13)	1504.58 (623.63)	0.016 *
Baseline WMH, log	Mean (SD)	1.77 (1.29)	1.68 (1.31)	1.77 (1.31)	1.85 (1.26)	0.764
Follow-up WMH, log	Mean (SD)	2.07 (1.37)	2.06 (1.30)	2.03 (1.40)	2.12 (1.43)	0.924
Change of WMH	Mean (SD)	0.31 (0.48) *** ^a^	0.37 (0.53) *** ^a^	0.25 (0.47) *** ^a^	0.28 (0.42) *** ^a^	0.337
Total grey matter volume, baseline, cm^3^	Mean (SD)	623.72 (58.65)	630.31 (56.56)	609.45 (64.68)	627.84 (54.87)	0.127
Mean thickness, baseline, mm	Mean (SD)	2.47 (0.15)	2.46 (0.15)	2.45 (0.15)	2.49 (0.15)	0.374
Gender						
Male	n (%)	89 (48.63%)	44 (65.67%)	17 (34.00%)	28 (42.42%)	0.001 **
Female	n (%)	94 (51.37%)	23 (34.33%)	33 (66.00%)	38 (57.58%)	
Race/ethnicity						
Non-Hispanic white and others	n (%)	120 (65.57%)	44 (65.67%)	34 (68.00%)	42 (63.64%)	0.650
Non-Hispanic black	n (%)	40 (21.86%)	12 (17.91%)	12 (24.00%)	16 (24.24%)	
Hispanic	n (%)	23 (12.57%)	11 (16.42%)	4 (8.00%)	8 (12.12%)	

* *p* < 0.05; ** *p* < 0.01; *** *p* < 0.001. Abbreviation: MeDi = Mediterranean diet; NARTIQ = National Adult Reading Test-assessed Intelligence Quotient; WMH = White matter hyperintensity. ^a^ A one sample *t*-test was used to determine if the change in WMH burden from baseline to follow-up equals zero over an average of five years.

**Table 2:** Association between Mediterranean diet and change in white matter hyperintensity by population groups.

	Model 1			Model 2			Model 3		
	β (95% CI)	*p*	*p*-Inter ^a^	β (95% CI)	*p*	*p*-Inter ^a^	β (95% CI)	*p*	*p*-Inter ^a^
MeDi score	−0.015 ** (−0.027–−0.002)	0.020	--	−0.014 ** (−0.026–−0.002)	0.028	--	−0.014 ** (−0.026–−0.001)	0.034	--
By age group									
Aged < 43 yrs (n = 51)	−0.038 *** (−0.057–−0.018)	0.0002	Ref.	−0.035*** (−0.056–−0.014)	0.002	Ref.	−0.035 *** (−0.058–−0.013)	0.003	Ref.
Aged 43–64 yrs (n = 72)	−0.002 (−0.020–0.024)	0.872	0.024 **	−0.005 (−0.032–0.022)	0.730	0.067 *	−0.004 (−0.032–0.023)	0.771	0.075 *
Aged ≥ 65 yrs (n = 60)	−0.002 (−0.021–0.017)	0.836	0.017 **	0.0001 (−0.018–0.018)	0.991	0.037 **	0.0003 (−0.018–0.019)	0.971	0.037 **
By gender									
Male (n = 89)	−0.007 (−0.024–0.010)	0.429	Ref.	−0.008 (−0.026–0.011)	0.401	Ref.	−0.006 (−0.024–0.013)	0.526	Ref.
Female (n = 94)	−0.022 ** (−0.041–−0.003)	0.025	0.332	−0.016 (−0.035–0.002)	0.088	0.618	−0.017 (−0.036–0.002)	0.071	0.624
By race/ethnicity									
Non-Hispanic White and other (n = 120)	−0.011 (−0.026–0.004)	0.154	Ref.	−0.015 ** (−0.030–−0.0003)	0.045	Ref.	−0.015 (−0.030–0.0002)	0.052	Ref.
Non-Hispanic Black (n = 40)	−0.009 (−0.037–0.019)	0.517	0.957	−0.006 (−0.039–0.028)	0.731	0.628	−0.008 (−0.043–0.027)	0.646	0.622
Hispanic (n = 23)	−0.020 (−0.060–0.019)	0.297	0.293	−0.007 (−0.056–0.041)	0.751	0.471	−0.007 (−0.060–0.047)	0.793	0.445
By MeDi group									
Low MeDi (n = 67)	−0.056 *** (−0.091–−0.020)	0.003	Ref.	−0.052 *** (−0.089–−0.015)	0.007	Ref.	−0.053 *** (−0.091–−0.015)	0.008	Ref.
Middle MeDi (n = 50)	−0.029 (−0.158–0.099)	0.647	0.769	−0.048 (−0.185–0.089)	0.481	0.938	−0.053 (−0.194–0.088)	0.451	0.913
High MeDi (n = 66)	0.001 (−0.031–0.033)	0.957	0.034 **	0.005 (−0.026–0.037)	0.737	0.050 **	0.007 (−0.026–0.039)	0.685	0.052 *

* *p* < 0.1; ** *p* < 0.05; *** *p* < 0.01. ^a^ *p*-inter indicates the *p* value of the interaction term of continuous MeDi score and age/gender/race(ethnicity)/MeDi group. Model 1 was adjusted by age and follow-up interval. Model 2 was additionally adjusted by gender, education, NARTIQ, race/ethnicity, total daily energy intake, and baseline WMH. Model 3 was additionally adjusted by baseline gray matter volume residual and baseline mean thickness. In each age group, age was not included in the adjustment; in each gender group, gender was not included in the adjustment; in each race/ethnicity group, race/ethnicity was not included in the adjustment.

**Table 3:** Association between individual food category and the change in white matter hyperintensity (log).

	Total Participants ^b^			Interaction of Age × Food ^c^		
	β	95% CI	*p*	β	95% CI	*p*-Inter
Cereal	−0.034	(−0.077–0.009)	0.122	−0.001	(−0.003–0.001)	0.368
Potato	0.024	(−0.029–0.076)	0.376	−0.001	(−0.004–0.002)	0.449
Fruit	0.022	(−0.031–0.075)	0.409	0.001	(−0.001–0.004)	0.338
Vegetable	−0.095	(−0.162–−0.028)	0.006	0.003	(−0.0002–0.007)	0.068
Legumes and nuts	−0.020	(−0.066–0.025)	0.378	0.001	(−0.001–0.004)	0.337
Fish	0.071	(−0.002–0.143)	0.056	0.001	(−0.004–0.005)	0.797
Olive Oil	0.009	(−0.037–0.055)	0.700	0.001	(−0.002–0.004)	0.677
Poultry ^a^	−0.036	(−0.083–0.012)	0.138	−0.0005	(−0.003–0.002)	0.705
Red meat ^a^	0.006	(−0.038–0.050)	0.790	0.001	(−0.001–0.004)	0.304
Dairy ^a^	−0.045	(−0.086–−0.004)	0.031	0.002	(−0.0001–0.004)	0.065
Alcohol ^a^	0.010	(−0.029–0.050)	0.609	0.003	(0.00005–0.005)	0.046

^a^ higher score indicated less intake. ^b^ All food categories were entered into models simultaneously, and the model was adjusted by age and follow-up interval. Each food category was estimated by scores of 0−5. ^c^ Age was modeled as a continuous variable. Each food category was in a separate model, and the models were adjusted by follow-up interval.

## Data Availability

The data presented in this study are available on request from the corresponding author.

## Supplementary Materials

The following supporting information can be downloaded at: https://www.mdpi.com/article/10.3390/nu14173664/s1, Table S1: Comparison of participants characteristics between those who were included and excluded in the final models, baseline; Table S2: The Mediterranean diet score and the frequency of consumption for each food category.
