# Supplementary material for: Mediterranean Diet and White Matter Hyperintensity Change over Time in Cognitively Intact Adults
Source: Nutrients. 2022 Sep 5;14(17):3664. doi: 10.3390/nu14173664 (PMC9460774; doi:10.3390/nu14173664)
Supplement: Supplementary file 1 [file nutrients-14-03664-s001.zip › nutrients-1843417-supplementary.pdf]

Table S1. Comparison of participants characteristics between those included and excluded in the final models baseline.

	Participants Included in the Analysis (n = 183)	Participants *Not* Included in the Analysis (n = 379)	*p*-Diff
Age, mean (SD), years	53.19 (16.52)	54.01 (17.28)	0.590
Sex			
Male, n (%)	89 (48.63)	154 (40.63)	0.073
Female, n (%)	94 (51.37)	225 (59.37)	
Education [a], mean (SD), years	16.33 (2.37)	16.08 (2.37)	0.243
NARTIQ [b], mean (SD)	117.82 (8.20)	116.38 (9.06)	0.071
Race/ethnicity [c]			
Non-Hispanic White and Others, n (%)	120 (65.57)	234 (62.07)	0.707
Non-Hispanic Black, n (%)	40 (21.86)	93 (24.67)	
Hispanic, n (%)	23 (12.57)	50 (13.26)	

[a] Three missing values are included in the "participants not included in the analysis" group. [b] Twenty-seven missing values are included in the "participants not included in the analysis" group. [c] Two missing values are included in the "participants not included in the analysis" group.

Table S2. The Mediterranean diet score and the frequency of consumption for each food category.

Score / Frequency of Consumption (Servings/Month)	0	1	2	3	4	5
Cereal	0	1–4	5–8	9–12	13–18	>18
Potato	0	1–6	7–12	13–18	19–22	>22
Fruit	0	1–6	7–12	13–18	19–22	>22
Vegetable	0	1–6	7–12	13–18	19–22	>22
Legumes and nuts	0	1–6	7–12	13–18	19–22	>22
Fish	0	1–6	7–12	13–18	19–22	>22
Oil	0	1–6	7–12	13–18	19–22	>22
Poultry	>22	19–22	13–18	7–12	1–6	0
Red meat	>22	19–22	13–18	7–12	1–6	0
Dairy	>22	19–22	13–18	7–12	1–6	0
Alcohol	0 & >60	31–60	15–30	5–14	3–4	1–2
